# Performance of the Ebel standard-setting method for the spring 2019 Royal College of Physicians and Surgeons of Canada internal medicine certification examination consisting of multiple-choice questions

**DOI:** 10.3352/jeehp.2020.17.12

**Published:** 2020-04-20

**Authors:** Jimmy Bourque, Haley Skinner, Jonathan Dupré, Maria Bacchus, Martha Ainslie, Irene W. Y. Ma, Gary Cole

**Affiliations:** 1 Exam Quality and Analytics Unit, Royal College of Physicians and Surgeons of Canada, Ottawa, ON, Canada; 2 Department of Medicine, University of Calgary, Calgary, AB, Canada; 3 Department of Medicine, University of Manitoba, Winnipeg, MB, Canada; Hallym University, Korea

**Keywords:** Canada, Certification, Medicine, Specialization, Standard-setting

## Abstract

**Purpose:**

This study aimed to assess the performance of the Ebel standard-setting method for the spring 2019 Royal College of Physicians and Surgeons of Canada internal medicine certification examination consisting of multiple-choice questions. Specifically, the following parameters were evaluated: inter-rater agreement, the correlations between Ebel scores and item facility indices, the impact of raters’ knowledge of correct answers on the Ebel score, and the effects of raters’ specialty on inter-rater agreement and Ebel scores.

**Methods:**

Data were drawn from a Royal College of Physicians and Surgeons of Canada certification exam. The Ebel method was applied to 203 multiple-choice questions by 49 raters. Facility indices came from 194 candidates. We computed the Fleiss kappa and the Pearson correlations between Ebel scores and item facility indices. We investigated differences in the Ebel score according to whether correct answers were provided or not and differences between internists and other specialists using the t-test.

**Results:**

The Fleiss kappa was below 0.15 for both facility and relevance. The correlation between Ebel scores and facility indices was low when correct answers were provided and negligible when they were not. The Ebel score was the same whether the correct answers were provided or not. Inter-rater agreement and Ebel scores were not significantly different between internists and other specialists.

**Conclusion:**

Inter-rater agreement and correlations between item Ebel scores and facility indices were consistently low; furthermore, raters’ knowledge of the correct answers and raters’ specialty had no effect on Ebel scores in the present setting.

## Introduction

### Rationale/background

To receive specialty certification in Canada, residents must pass the Royal College of Physicians and Surgeons of Canada’s (RCPSC) certification exam. In such a high-stakes context, a rigorous and legally defensible standard-setting procedure is essential to support the validity of inferences based on performance in subsequent decision-making [1]. Standard-setting can be defined as “the process of establishing one or more cut scores on examinations. The cut scores divide the distribution of examinees’ test performances into 2 or more categories” [2]. It is generally agreed that in the context of certification assessments, absolute standards, expressed as a number or percentage of test questions or marks, are preferable to relative standards [3]. Many methods are available for setting standards, including the Angoff, Ebel, Nedelsky, and Hofstee methods [2]. In the Ebel method, judges review test items and provide judgments about the minimum level of performance required to pass [4,5]. Unlike the well-studied Angoff method, scant empirical data have been published on the performance of the Ebel method in certification examinations. Moreover, the training required to rate items adequately might be perceived as burdensome, especially in cases where volunteers with limited time to spare perform the rating. We elected to focus on the Ebel method because it involves making judgments not only on item difficulty, but also on item relevance.

### Objectives

The present study aimed to examine the performance of the Ebel method for standard-setting on the multiple-choice question (MCQ) component of the RCPSC internal medicine certification examination in the context of minimal rater training. More precisely, we investigated inter-rater agreement between judges’ classification of test items; evaluated the correlations between item Ebel scores and empirical item facility indices, sought to determine whether the Ebel scores were modified by the provision of correct answers, and investigated whether the judges’ specialty influenced their rating scores and inter-rater agreement.

## Methods

### Ethics statement

We received ethical approval for this study from Université de Moncton (certificate number 1819-061). The requirement to obtain informed consent was exempted because this study involved an analysis of test results.

### Study design

This study presents an analysis of test results.

### Subjects

Ebel classification data come from the spring 2019 RCPSC certification exam in internal medicine. The judges were raters for the spring 2019 RCPSC internal medicine certification exam. Forty-nine raters out of a possible total of 62 (participation rate, 79%) applied the Ebel method to the 203 MCQ items.

### Technical information

The Ebel standard-setting method is an absolute standard-setting method that involves expert judgments along 2 dimensions—difficulty and relevance to the assessment of clinical competence—for each test item. In its implementation, the following 6 steps were completed: (1) Selection of judges: Forty-nine judges volunteered to apply the Ebel method. All the raters were volunteer physicians with current professional expertise and experience of practicing internal medicine in Canada. All the judges were asked to rate each of the 203 MCQ items on its difficulty and relevance. (2) Judges’ training: Prior to rating the items, we gave a 20-minute presentation about the purpose and process of the Ebel method. Specifically, the presentation explained how items were to be rated and clarified the meaning of difficulty and relevance in the context of a certification examination for internal medicine. We also used the presentation to introduce the study and obtain consent from the judges. Although the time requirements were considerably less than what would be recommended, the time required for both training (35 minutes) and rating (3 hours on average) strained the raters in terms of available time. (3) Definition of “minimally competent” performance: After the presentation, we discussed the expectations for minimally competent candidates with the judges for 15 minutes. (4) Data collection: Judges were seated in front of a computer. They entered relevance (essential, important, acceptable, or minimal) and difficulty (easy, medium, or hard) ratings for each of the 203 MCQ items, using drop-down menus in a Microsoft Excel spreadsheet. The process took approximately 3 hours, on average. The exam consisted of 2 booklets. For each judge, the correct answers to the MCQ questions were provided for one randomly chosen booklet, but not the other. From our experience with other specialties, we observed that the use of individual ratings—instead of consensus ratings—avoided the pitfall of polarizing ratings around the values suggested by more vocal examiners. (5) For each item category (e.g., essential and easy), judgments were collected on the proportion of marks or items expected to be correctly answered by minimally competent candidates. To save time and to reduce the burden on judges, we used the grid proposed by Ebel [4] and used recently by Park et al. [6] instead of having judges estimate the success rate for each item (Table 1). However, as Ebel ratings are context-specific, anecdotal evidence from Ebel ratings with other specialties, based either on rater estimates or previous facility indices, suggested that the values in the grid were somewhat low for a certification test. The resulting Ebel score might, therefore, have underestimated the acceptable cut score. (6) Computation of the Ebel score: Next, we calculated Ebel scores for each item and for the test as a whole as the average of the scores given by each judge, separately with regard to whether or not the correct answers were provided. Empirical facility indices (difficulty index) were then added from the exam database.

### Statistical analysis

We computed empirical facility indices for all MCQ items based on the performance of 194 candidates who took the MCQ component of the examination in the spring of 2019 and were defined as “minimally competent” (i.e., just competent enough for unsupervised practice). The categorization as minimally competent was operationalized as an exam score falling within 1 standard error of measurement (SEM) of the unadjusted pass score of 70% (SEM=4.98). The correlations of item facility indices with the Ebel scores set by judges were then analyzed. We quantified inter-rater agreement using the Fleiss kappa with the Realstats add-in for Microsoft Excel [7]. Interpretation followed guidelines of Landis and Koch [8], with kappa values of 0–0.20 considered to indicate slight agreement, 0.21–0.40 fair agreement, 0.41–0.60 moderate agreement, 0.61–0.80 substantial agreement, and 0.81–1.00 almost perfect agreement. We evaluated correlations between item Ebel scores and the empirical item facility indices using Pearson product-moment correlation coefficients. We used the paired-sample t-test and item statistics to investigate differences in Ebel scores according to whether or not raters were provided with the correct answers. We conducted the independent-sample t-test to compare Ebel ratings between judges who were general internists and other specialists. All analyses, except for the Fleiss kappa calculations, were carried out with IBM SPSS ver. 26.0 (IBM Corp., Armonk, NY, USA). Raw data were available from Dataset 1.

## Results

### 1. Inter-rater agreement

Variation was observed in ratings for both difficulty and relevance. The Fleiss kappa among the 49 raters was 0.10 (95% confidence interval [CI], 0.10–0.11) for difficulty and 0.11 (95% CI, 0.11–0.12) for relevance, which corresponded to slight agreement [8]. To evaluate the potential effects of rater fatigue or boredom [9,10], we assessed inter-rater agreement for only the first of the 2 booklets (101 items). Agreement on the first booklet was not considerably different than that on all 203 items (κ=0.11; 95% CI, 0.11–0.12 for difficulty; κ=0.11; 95% CI, 0.10–0.11 for relevance). Furthermore, considering only ratings from booklet 1 for which the correct answers were provided (25 raters) to reduce noise also did not convincingly improve the ratings (κ=0.14; 95% CI, 0.14–0.15 for difficulty; κ=0.11; 95% CI, 0.10–0.12 for relevance).

### 2. Correlation between Ebel scores and item facility indices

**Fig. 1.**
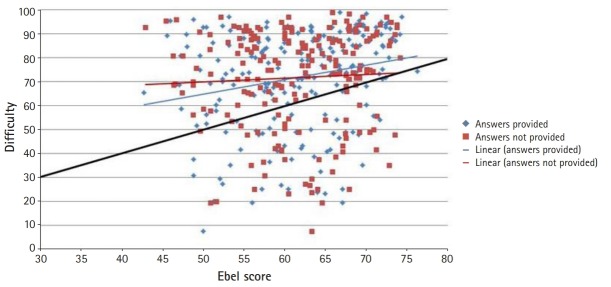
Correlation between Ebel scores and item facility indices.

The correlation between item Ebel scores and empirical item facility indices was low when correct answers were provided (r=0.22; P=0.002; 95% CI, 0.08–0.35) and negligible when they were not (r=0.05; P=0.445; 95% CI, -0.09 to 0.19) (Fig. 1).

**Fig. 2.**
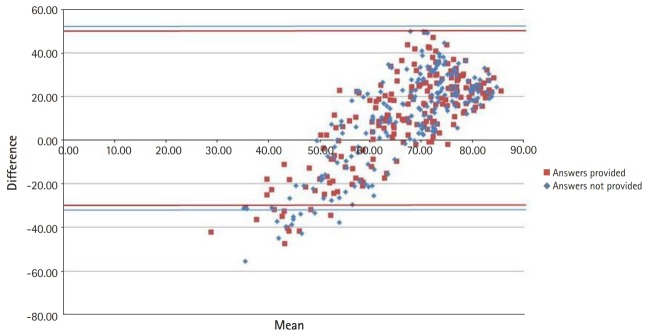
Bland-Altman plot of the difference between item facility indices and Ebel scores.

Fig. 1 illustrates the high concentration of item facility indices between 0.70 and 1.00. The black diagonal line indicates equality between the Ebel score and empirical item facility. Very few items fell directly on or around that line, revealing substantial discrepancies between the Ebel scores and item facility indices, with the overall test being generally easier than the Ebel ratings suggested. Both trend lines showed a positive slope that was less than 1, revealing a tendency for judges to overestimate the difficulty of easy items and to underestimate the difficulty of hard items, as further illustrated in Fig. 2. The Bland-Altman plot showed that the difference between item facility indices and Ebel scores was negative for the few harder items (with facility indices up to about 0.60), and positive for the easier items (Fig. 2).

When evaluating the correlations between item facility indices and each of the 2 Ebel ratings separately, we would expect a high, positive, statistically significant correlation with the difficulty rating and a lower correlation with the relevance rating, since it measures a slightly different construct. However, although positive, both correlations were low and non-significant (r=0.12; P=0.092; 95% CI, -0.02 to 0.25 and r=0.11; P=0.128; 95% CI, -0.03 to 0.24, respectively). The 2 components, however, were highly correlated with each other (r=0.70; P<0.001; 95% CI, 0.62–0.76), suggesting that judges might not have been able to disentangle the 2 constructs consistently when rating items.

### 3. Effect of correct answer availability on the Ebel score

The Ebel score for the whole exam was practically the same (61.5) regardless of whether the correct answers were available to the raters (Table 2).

### 4. Effect of raters’ specialty on the Ebel score and inter-rater agreement

We hypothesized that general internal medicine specialists, being a more homogeneous group, would show greater inter-rater agreement than other specialists and that their Ebel score would be different and more accurate because of the greater congruence between their expertise and the exam content. However, inter-rater agreement was of the same order of magnitude for both groups (Table 3), and the Ebel scores given by general internal medicine specialists (61.4; 95% CI, 60.0–62.6) did not differ significantly (t[47]=0.35; P=0.727; d=0.10) from those given by other specialists (61.7; 95% CI, 60.0–63.4).

## Discussion

The context in which we applied the Ebel method was typical of Royal College exam development meetings, in that the rating was performed by volunteers on a very tight schedule, which necessitated reducing training time to a minimum. The Ebel standard-setting method appeared to be easy to implement and apply while being generally accepted as valuable by raters.

### Interpretation

The correlations between Ebel scores and item facility indices were negligible and failed to reach statistical significance, even when only considering the difficulty component ratings. In the literature, the correlations between Ebel scores and item facility indices have been reported to be around 0.40 (range, 0.25–0.60) on average [11], which is much higher than the correlations that we observed. One possible explanation for this discrepancy is that the low variability in item facility indices, with the majority falling between 0.70 and 1.00, attenuated the correlation. Another possible reason for the low correlations between Ebel scores and item difficulty indices is the considerable variation and slight (κ<0.20) agreement between raters for both item difficulty and relevance. We did not find many evaluations of inter-rater agreement in the literature. Although Downing et al. [12] observed that the intra-rater consistency measured by intra-class correlations was over 0.75 for Ebel ratings, their results did not provide information on the degree of consensus regarding these ratings among multiple raters. In another study, Swanson et al. [13] pointed out considerable variation in inter-rater reliability, with intra-class correlations between 0.09 and 0.79. In our case, inter-rater agreement remained low even when taking into account fatigue and boredom [9,10] and whether raters were provided with the correct answers.

Moreover, our analyses detected no discernable differences in inter-rater agreement and Ebel scores between general internists and other specialists. This finding is a counterintuitive outcome. It raises the question as to whether there would be differences if we were to take item content into account. More precisely, future research could explore whether the concordance (or lack thereof) between raters’ specialty and item content makes a difference in inter-rater agreement for difficulty and relevance ratings, as well as in Ebel scores. Future research should look into whether clearer and more thorough definitions, more extensive rater training, and rater calibration before the application of the Ebel method could improve inter-rater agreement. However, the issue might then become the applicability of the method given the extensive training required.

The effect, if any, of knowing the correct answer before rating the item has not been explored yet in the literature on the Ebel method, although it is a relevant detail that could have repercussions on the way the method is implemented. Our results suggest that having access to the correct answer did not result in a significant difference in the Ebel score. Therefore, both options seem viable when implementing the Ebel method.

The results of the present study also corroborate a sort of regression towards the mean documented by other authors [14,15], where judges underestimated the difficulty of hard items and overestimate that of easy ones. This may have been partially caused by the value limitations in the Ebel grid, in which the Ebel scores were constrained to an interval from 0.30 to 0.80. It follows that further research should focus on the relative merits of different ways of deriving grid values, such as the values published by Ebel [4], estimates by judges, and the use of empirical values from past exams.

### 1. Limitation

Although disappointing, the results are not entirely surprising since, in the scientific literature, expert raters have generally been found to be poor at judging the difficulty of items, even with normative data available, regardless of the particular standard-setting method that is used [14]. In our case, the minimal training offered to raters was probably a compounding factor.

### 2. Conclusion

The above finding of slight inter-rater agreement should be overcome by more in-depth training of raters. It may be possible to correct the low correlation between item Ebel scores and item facility indices by producing items with more variable facility indices. There is no need to inform raters of the correct answers because knowledge of the correct answers had no effect on the Ebel score. The absence of an impact of raters’ specialty on rating scores and inter-rater agreement were favorable findings regarding the possibility of recruiting raters from a variety of specialties.

## Figures and Tables

**Table 1.** Ebel grid

Relevance	Difficulty		
	Easy	Medium	Hard
Essential	80	70	60
Important	70	60	50
Acceptable	60	50	40
Minimal	50	40	30

**Table 2.** Descriptive statistics for item Ebel scores (N=203)

	Correct answer provided	Correct answer not provided
Ebel score	61.5	61.5
95% Confidence interval	60.5–62.5	60.5–62.5
Standard deviation	7.5	7.1
Median	62	61.7
Minimum	42.7	42.9
Maximum	76.3	74.2

The paired-sample t-test was used to corroborate the absence of a significant difference between the 2 sets of scores (t[202]=0.10; P=0.923; d=0.00).

**Table 3.** Inter-rater agreement for internists and other specialists

	Difficulty	Relevance
General internists	0.12 (95% CI, 0.11–0.12)	0.11 (95% CI, 0.11–0.12)
Others	0.07 (95% CI, 0.06–0.07)	0.08 (95% CI, 0.08–0.09)

CI, confidence interval.

## Footnotes

**Data availability** Data files are available from https://doi.org/10.7910/DVN/OGZ7CL Dataset 1. Data file and data dictionary.

## Supplementary materials

Supplement 1. Audio recording of the abstract.
